# Zebrafish and *Galleria mellonella*: Models to Identify the Subsequent Infection and Evaluate the Immunological Differences in Different *Klebsiella pneumoniae* Intestinal Colonization Strains

**DOI:** 10.3389/fmicb.2019.02750

**Published:** 2019-12-02

**Authors:** Xiucai Zhang, Yajie Zhao, Qing Wu, Jie Lin, Renchi Fang, Wenzi Bi, Guofeng Dong, Jiahui Li, Yizhi Zhang, Jianming Cao, Tieli Zhou

**Affiliations:** ^1^Department of Clinical Laboratory, The First Affiliated Hospital of Wenzhou Medical University, Wenzhou, China; ^2^School of Laboratory Medicine and Life Science, Wenzhou Medical University, Wenzhou, China; ^3^Department of Clinical Laboratory, The Fourth Affiliated Hospital of Zhejiang University School of Medicine, Yiwu, China

**Keywords:** *Klebsiella pneumoniae*, intestinal colonization, zebrafish, infection, *Galleria mellonella*

## Abstract

The intestine is the main reservoir of bacterial pathogens in most organisms. *Klebsiella pneumoniae* is an important opportunistic pathogen associated with nosocomial bacterial infections. Intestinal colonization with *K. pneumoniae* has been shown to be associated with an increased risk of subsequent infections. However, not all *K. pneumoniae* strains in the intestine cause further infection, and the distinction of the difference among strains that cause infection after colonization and the ones causing only asymptomatic colonization is unclear. In this study, we report a case of a hospitalized patient from the ICU. We screened out two intestine colonization strains (FK4111, FK4758) to analyze the subsequent infection conditions. We set up infection models of zebrafish and *Galleria mellonella* to establish the differences in the potential for causing subsequent infection and the immunological specificities after *K. pneumoniae* intestine colonization. Sudan Black B and neutral red staining results indicated that FK4758 was more responsive to neutrophil recruitment and phagocytosis of macrophages than FK4111. The results of the assessment of the organ bacterial load revealed that FK4111 and FK4758 both had the highest bacterial loads in the zebrafish intestine compared to those in other organs. However, in the zebrafish spleen, liver, and heart, the FK4758 load was significantly higher than that of FK4111. The ST37 strain FK4111, which does not produce carbapenemase, did not cause infection after colonization, whereas the ST11 strain FK4758, which produces carbapenemase, caused infection after intestinal colonization. Our finding demonstrated that not all intestinal colonization of *K. pneumoniae* subsequently caused infections, and the infections of *K. pneumoniae* after colonization are different. Therefore, the infection models we established provided possibility for the estimation of host-microbial interactions.

## Introduction

High medical expenses and considerable morbidity are usually associated with the occurrence of infection in the intensive care unit (ICU). This expenditure accounts for approximately 40% of the total ICU expenditure and infections have become the main cause of death in ICU (Freedberg et al., 2018). Patients in ICU are more likely to acquire pathogen infections while hospitalized than other patients, with an attributable mortality of up to 25% (Vincent et al., 2009). *Klebsiella* spp. is one of the most common causes of health care-associated infections, which caused approximately 10% of all hospital-acquired infections (Magill et al., 2014). Among them, *Klebsiella pneumoniae* is one of the main pathogens that cause hospital-acquired urinary tract infections, pneumonia, hematosepsis, and soft tissue infection (Podschun and Ullmann, 1998). More seriously, *K. pneumoniae* can colonize the intestine, entering into other tissues through the intestine, where it leads to severe infections, such as human bloodstream infection (Paczosa and Mecsas, 2016). *K. pneumoniae* colonization has been confirmed as an important risk factor for ICU infection (Hsu et al., 2015; Gorrie et al., 2017). Hsu et al. published a paper in which they described the interactions between intestinal epithelial cells and clinical *K. pneumoniae* strains causing systemic infections (Hsu et al., 2015). Another recent study tested the hypothesis that intestinal colonization leads to subsequent infection with *K. pneumoniae* in hospitalized patients (Martin et al., 2016). However, not all *K. pneumoniae* strains colonized in the intestine cause further infection; the majority of patients with colonized pathogens did not suffer from infections for several years or even decades (Harada et al., 2011). Few reports exist on the experimental basis of colonization and infection. Moreover, it is still unclear how to distinguish between strains that cause infection after colonization and those that lead to only asymptomatic colonization.

Most of the studies on *K. pneumoniae* have been conducted in animal models such as mice, rats, and pigs. However, these mammalian models have many limitations: (1) expensive maintenance and operational facilities; (2) the possibilities for real-time analysis are limited; and (3) ethical and practical limitations are present (Marcoleta et al., 2018). Therefore, it has become an urgent problem to find a more suitable host animal model to study intestinal colonization. In recent years, a large number of classical infection models of mammals have been established (Paczosa and Mecsas, 2016), such as *Galleria mellonella* (Wand et al., 2013), *Caenorhabditis elegans* (Kamaladevi and Balamurugan, 2017), and zebrafish (Cheepurupalli et al., 2017). Those models have been successfully applied for virulence testing, infection-pathogenesis, and antimicrobial/antiviral drugs screening.

Zebrafish is an ideal vertebrate host model, whose genome is highly similar to that of humans (>87%) (Du et al., 2016). In addition, zebrafish harbors a mammalian-like innate immune system, and at the early developmental stages of embryos, innate immunity includes mainly macrophages and neutrophils. These cells have been confirmed to be the first responders to invading pathogens (Podschun and Ullmann, 1998). Macrophages and neutrophils *in vivo* accumulated and migrated after stimulation by foreign pathogens in zebrafish during the optically transparent early life stages, and the host response to infection could be directly measured in real time (Renshaw et al., 2006). Zebrafish also have many other advantages, for instance, transparent body in the early life stages, easy propagation, and rapid growth (Meeker and Trede, 2008). Based on the above advantages, the model is suitable for research on host-pathogen interactions, the related immune responses, and the bacterial clearance (Kanther and Rawls, 2010; Renshaw and Trede, 2012). In particular, zebrafish is considered to be a new model for *in vivo* studies of bacterial interactions with neutrophils and phagocytes (Wu et al., 2016). It has been reported that zebrafish infection model was successfully used in *Mycobacterium marinum* (Adams et al., 2011), *Salmonella typhimurium* (Wu et al., 2016), and *Staphylococcus aureus* (Li and Hu, 2012) to investigate the pathogenesis of infection. In addition, adult zebrafish have been proven to be a valuable model of infectious disease for the evaluation of organ bacterial load (Vojtech et al., 2009). Thus, zebrafish have been increasingly used as a model for investigations on pathogen infection.

In this study, we report a case of a hospitalized patient from the ICU of the First Affiliated Hospital of Wenzhou Medical University, China. Eleven *K. pneumoniae* strains were isolated from the patient samples during the period of hospitalization. One was isolated from the stool samples; it caused bloodstream infection 10 days later, leading to the death of the patient. Prior to this, *K. pneumoniae* had also been detected in the patient’s stool samples but it did not cause infection. That phenomenon attracted our research interest and attention. We speculated that the *K. pneumoniae* that colonized in the intestines 10 days before the death of the patient could cause subsequent infection, whereas other colonization bacteria could not cause such infection. Therefore, using a zebrafish infection mode, we aimed to establish the differences in the potential for causing subsequent infection and the immunological specificities after *K. pneumoniae* intestine colonization. To the best of our knowledge, this is the first report of using zebrafish to determine the distinction between *K. pneumoniae* colonization and infection.

## Materials and Methods

### Collection of Clinical Isolates and Patient Information

From June 2017 to January 2018, a total of 11 *K. pneumoniae* strains were isolated from a hospitalized patient in the ICU of the first Affiliated Hospital of Wenzhou Medical University (Wenzhou, Zhejiang, China). The bacterial strains used in this work are listed in Table 1. FK4111 and FK4758, isolated from stool specimens, were considered to colonize the patient’s intestine and were used for subsequent animal colonization and the development of infection models. The identification of the isolates was performed using a VITEK 2 system (bioMérieux, Marcy L’Etoile, France). Patient information was obtained from the electronic medical records. This study and its consent procedure were approved by the Ethical Committee of the first Affiliated Hospital of Wenzhou Medical University. As the study was retrospective, and participants were anonymized, informed consent was not required.

**Table 1:** Susceptibility to the antimicrobial agents, basic characterization, and phenotypic detection of carbapenemase.

Strain ID	ST type	Source	Isolation date	MIC(μg/ml)										mCIM
				GEN	TOB	CAZ	CRO	LVX	CIP	IMP	ETP	PB	TCL	
FK4038	ST11	Sputum	06/02/17	>128	>128	>128	>64	64	>64	16	>64	2	2	+
FK4109	ST11	Wound	06/22/17	>128	>128	>128	>64	64	64	32	>64	1	2	+
FK4111^a^	ST37	Stool	06/23/17	>128	>128	>128	64	32	64	8	>64	0.5	2	−
FK4134	ST11	Urine	06/30/17	>128	>128	>128	>64	64	64	32	>64	0.25	2	+
FK4139	ST11	Sputum	06/30/17	>128	>128	>128	>64	64	64	32	>64	>32	1	+
FK4154	ST11	Sputum	07/06/17	>128	>128	>128	>64	64	64	32	>64	0.5	2	+
FK4271	ST11	Sputum	08/11/17	>128	>128	>128	>64	64	>64	32	>64	0.25	2	+
FK4431	ST11	Sputum	09/24/17	>128	>128	>128	>64	64	64	16	>64	0.25	1	+
FK4623	ST37	Stool	11/17/17	64	4	>128	>64	4	4	32	64	0.125	2	−
FK4758^a^	ST11	Stool	01/01/18	>128	>128	>128	>128	64	128	16	>128	0.5	2	+
FK4797	ST11	Blood	01/13/18	>128	>128	>128	>128	64	128	8	>128	1	2	+

*MIC, minimum inhibitory concentration; GEN, gentamycin; TOB, tobramycin; CAZ, ceftazidime; CRO, ceftriaxone; LVX, levofloxacin; CIP, ciprofloxacin; IMP, imipenem; ETP, ertapenem; PB, colistin; TCL, tigecycline; mCIM, modified carbapenem inactivation test*.

a *Isolates were considered to colonization in the patient’s intestinal, and used for subsequent animal colonization and infection models*.

### Antimicrobial Susceptibility Testing and Phenotypic Detection of Carbapenemase

The minimum inhibitory concentrations (MICs) of tested antibiotics (gentamycin, tobramycin, ceftazidime, ceftriaxone, levofloxacin, ciprofloxacin, imipenem, and ertapenem) were performed by the agar dilution method based on the Clinical and Laboratory Standards Institute (CLSI) guidelines (Clinical and Laboratory Standards Institute, 2018). Furthermore, the MICs for colistin and tigecycline were determined using the broth microdilution method and the results were interpreted according to the EUCAST breakpoints (available at http://www.eucast.org/clinical_breakpoints/). *Escherichia coli* ATCC 25922 and *Pseudomonas aeruginosa* ATCC 27853 were used for quality control. Furthermore, detection of carbapenemase-producing *K. pneumoniae* was performed by the modified carbapenem inactivation (mCIM) tests following the CLSI guidelines.

### Homology Analysis of Isolates

Multilocus sequence typing (MLST) of 11 *K. pneumoniae* isolates was carried out by amplification of the seven housekeeping genes (*gapA*, *infB*, *mdh*, *pgi*, *phoE*, *rpoB,* and *tonB*). Allelic profiles and sequence types (STs) were determined using the database available at the *K. pneumoniae* MLST website.^1^

Further, the genetic relatedness of the 11 strains isolated from the patient was determined by pulse field gel electrophoresis (PFGE). The method was applied based on previous reports with slight modifications (Muratani et al., 2006). Briefly, genomic DNA was digested by the *XbaI* (TaKaRa, Japan), then electrophoresis was performed under suitable conditions (temperature 14°C, voltage 6 V/cm, pulse angle 120°, and pulse duration of 5–35 s, maintained 18.5 h). The universal standard strain *Salmonella enterica* serotype H9812 was used as the size marker. PFGE results were interpreted in compliance with the international recommendations (Tenover et al., 1995). The results were further analyzed using Quality One software (BioRad Laboratories, USA). The dendrogram was built with the UPGMA (unweighted pair group using arithmetic averages) hierarchical algorithm.

### Zebrafish

The zebrafish wild-type AB is maintained at the Zebrafish Culture Center of Wenzhou Medical University. Zebrafish embryos were obtained by natural spawning of the wild type and raised in petri dishes containing E3 medium and 0.1% methylene blue until 3 days post-fertilization (dpf). Adult wild-type zebrafish (above 6 months old) were reared in recirculation systems under standard conditions at 28.5°C. All zebrafish experiments were carried out following protocols approved by the Institutional Animal Care and Use Committee (IACUC) at Wenzhou Medical University.

### Assessment of Intestinal Colonization Strains on Host Phagocytes Responses

*K. pneumoniae* strains were routinely grown at 37°C with shaking (180 rpm) in Luria-Bertani broth (10 g/L triptone, 5 g/L yeast extract, 5 g/L NaCl) until their exponential phase of growth (OD_600_ = 0.4–0.6) was reached. The concentration of strain suspensions applied was based on the results of previous similar studies (Marcoleta et al., 2018) and preliminary laboratory experiments. Intestinal colonization was simulated by immersing zebrafish larvae (naturally hatched zebrafish at 3 days post fertilization (dpf)) in cultured strain suspensions (FK4111 and FK4758).

Sudan Black B is a lipid staining agent with specifically dyeing particles in neutrophils. After zebrafish was co-cultured with bacteria for 8 hours, 10 zebrafish were taken from each experimental group (FK4111, FK4758, and control). The zebrafish were then fixed with 4% paraformaldehyde, then rinsed with PBST and stained with Sudan black B (Solarbio, USA), staining specifically neutrophils. The neutrophils recruitment was observed under a microscope after washing with 70% ethanol. The specific configuration steps of the dye solution were performed as described in a previous study (Wu et al., 2016).

To evaluate macrophages phagocytosis, 10 larvae from each of the two groups infected with FK4111 and FK4758 were respectively cultured in a 12-well plate containing 2.5 mg/L of neutral red (Solarbio, USA) dye solution. The neutral red dye can aggregate into the macrophages forming red dot aggregates due to the endocytosis of macrophages (Ma et al., 2015). After co-incubation of zebrafish with bacteria for 8 h, the larvae were anesthetized and the phagocytosis of neutral red by the macrophages was observed under a microscope.

### Assessment of Bacterial Burden of Organs After Intestinal Colonization

**Figure 1:**
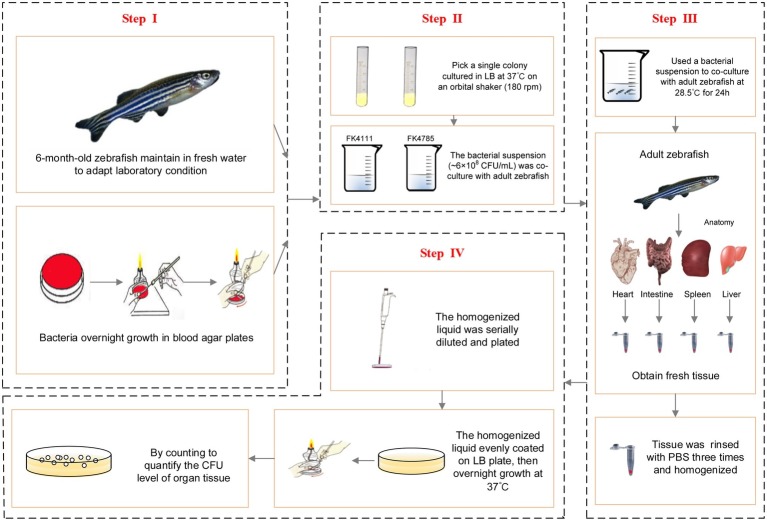
Protocol of bacterial infection and metastasis assessment.

In order to further establish whether FK4111 and FK4758 could cause infection after colonization, we used 6-month-old zebrafish as a model for bacterial immersion experiments, and then dissected different zebrafish organs for bacterial counting. Immersion assays for *K. pneumoniae* were adapted from similar methods performed in previous studies (Marcoleta et al., 2018). The OD_600_ and growth conditions of bacterial strains were same as those used in the assessment of intestinal colonization strains in the host phagocytes responses. Based on the results of our pre-experiments and other related research, we used a bacterial suspension (approximately 6 × 10^8^ CFU/ml) for co-culture with adult zebrafish for 24 h. The intestines, spleen, liver, and heart of 10 zebrafish individuals from each group were obtained and subjected to bacterial isolation. The tissue was homogenized using a hand-held tissue homogenizer in 2 ml of sterile phosphate buffered saline (PBS) for 20 s, then rinsed with 1 mL of PBS, and the homogenized liquid was serially diluted and plated to quantify the CFU level of the organ tissue. The protocol developed for bacterial infection and metastasis assessment is illustrated in Figure 1.

### *In vivo* Studies: *Galleria mellonella* as an Infection Model

*G. mellonella* infection assays were performed as described previously, with slight modifications (Wand et al., 2011). We used *G. mellonella* larvae to evaluate the difference of virulence between FK4111 and FK4758. All experimental reagents and equipment have been disinfected before the experiment. Overnight cultures of *K. pneumoniae* strains were adjusted using PBS with concentrations of 1 × 10^6^ CFU/ml. After surface disinfection using ethanol (70%), larvae were injected with 10 μl of bacterial suspension using a Hamilton precision syringe (Tsai et al., 2016). Uninfected larvae (either uninoculated or injected with 10 μl of phosphate buffered saline) were used as controls. We sterilized the Hamilton precision syringes with 70% alcohol after each use, and washed it three times with sterile water before the next injection. The plates containing the *G. mellonella* were incubated at 37°C in the dark. The survival of larvae was counted at 24-h intervals for 3 days. All experiments were performed in triplicate. Statistical analysis was performed using the Kaplan–Meier method.

### Statistical Analysis

The results were compared using the Student’s *t*-test. Difference was considered significant at a value of *p* below 0.05. All statistical analyses were performed using GraphPad Prism version 6.0.

## Results

### Case Description

In June 2017, a 69-year-old male patient, with complaints of fever and cough for over 2 months, was admitted to the First Affiliated Hospital of Wenzhou Medical University, Wenzhou, China. The patient had undergone brain stem tumor resection 1 year ago. He developed symptoms such as fever and cough for 2 months, during which septic shock occurred, causing unconsciousness. Then, he was admitted to the ICU, and treated with cefoperazone-sulbactam, dispersibleco-trimoxazole, meropenem, tigecycline and fosfomycin piperacillin, and tazobactam. Invasive treatment was also performed, such as endotracheal intubation and enteral feeding tube. During hospitalization, 11 *K. pneumoniae* strains were isolated from multiple specimens (sputum, wound, stool, urine, and blood). Among them, FK4111, FK4623, and FK4758 were separated from stool, and the ST type determined were ST37, ST37, and ST11, respectively. It was worth noting that in January 2018, 10 days after the date of isolation of ST11 *Klebsiella pneumoniae* from the patient’s stool, the patient developed a bloodstream infection. In June and November 2017, ST37 *K. pneumoniae* also colonized the intestine but did not cause infection. Death occurred after the patient had bloodstream infection (Supplementary Figure S1).

### Antimicrobial Susceptibility and Phenotypic Detection of Carbapenemase

According to the results of the antimicrobial susceptibility test, all the strains of *K. pneumoniae* were multidrug resistant. Furthermore, the mCIM results showed that all the strains were negative except for two strains (FK4111 and FK4623) (Table 1).

### Homology Analysis of the Isolates

**Figure 2:**
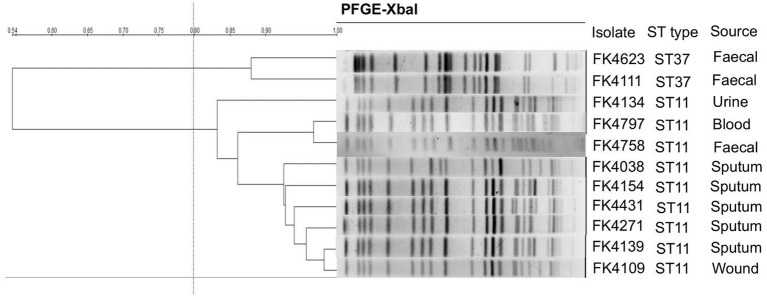
Pulsed-field gel electrophoresis (PFGE) patterns and multilocus sequence typing (MLST) for the 11 *K. pneumoniae* isolates. QualityOne software (Bio-Rad Laboratories, USA) was used to analyze the relatedness. The phylogenetic tree was generated by UPGMA clustering.

The clonal correlation of 11 multidrug-resistant (MDR) strains isolated from different specimens was studied by MLST and PFGE. Two distinct MLST sequence types were observed among the 11 isolates, including ST37 (2/11, 18.2%) and ST11 (9/11, 81.8%). Among the three strains isolated from stool, two strains (FK4111, FK4623) belonged to ST37, and one strain (FK4758) was ST11. According to the PFGE patterns, two clusters were observed, one contained two ST37 strains and another contained nine ST11 strains. As shown in Figure 2, two ST37 strains belonged to the same cluster, whereas the remaining nine ST11 strains belonged to another cluster.

### Neutrophils Recruitment to Infection Loci in Zebrafish Larvae

**Figure 3:**
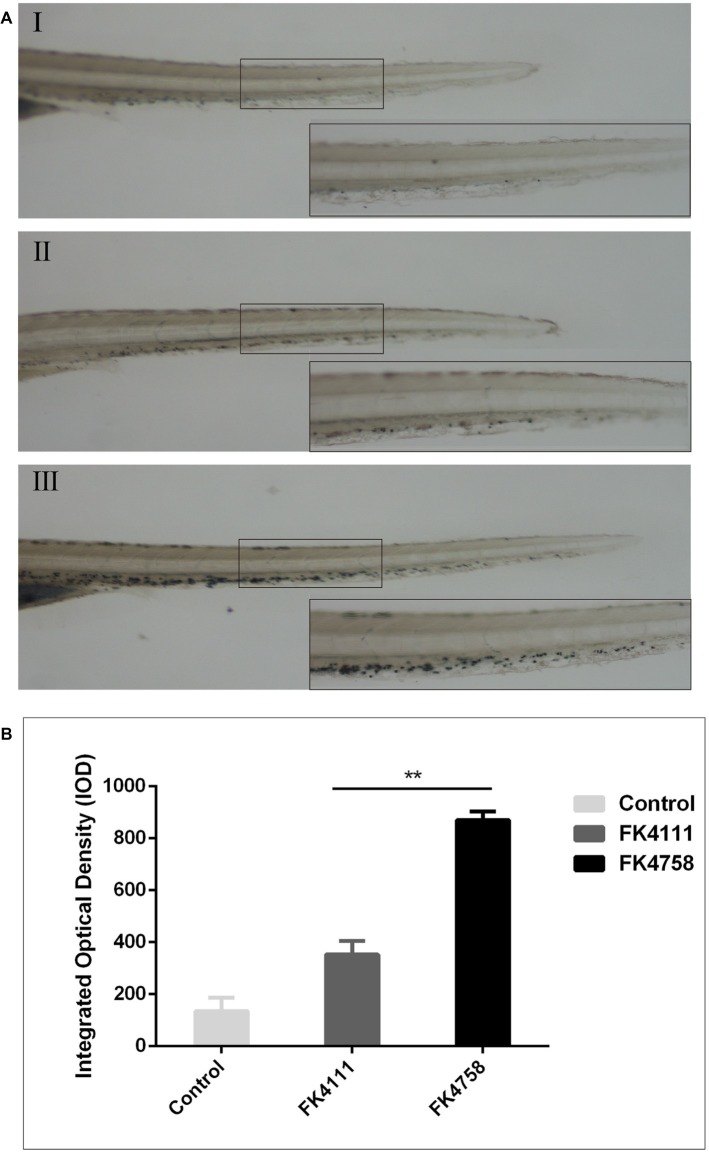
Impact of different colonization strains on neutrophils recruitment. **(A)** Zebrafish larvae at 3 dpf were immersed in water containing bacteria with a final concentration of 6 × 10^8^ CFU/ml, and detected by Sudan black B staining at 8 hpi (*n* = 10/group). The bottom right corner (100×) is an enlarged figure of the black box in the left (40×). (I) Control group; (II) the group of FK4111 infection; (III) the group of FK4758 infection. **(B)** Quantitative analysis of recruitment of neutrophils using integral optical density (IOD) by Image-Pro Plus image analysis software version 6.0. ^**^p < 0.01.

As evident in Figure 3A, the black particles formed in neutrophils could be clearly observed under the stereoscopic microscope. In the control group, no obvious black granules were formed by neutrophil recruitment in zebrafish. After immersion colonization with the two strains (FK4111 and FK4758) of *K. pneumoniae*, black granules appeared in varying degrees, suggesting difference in the neutrophils recruitment. Significantly more black granules were available in the FK4758-infected group than in the control and FK4111 groups. We also quantitatively analyzed the recruitment of neutrophils using integral optical density (IOD); the difference between FK4111 and FK4758 was statistically significant (*p* < 0.05). However, no significant difference was found between strain FK4111 and the control group in the formation of black granules (*p* > 0.1), as shown in Figure 3B.

### Macrophages Phagocytosis in Zebrafish Larvae

**Figure 4:**
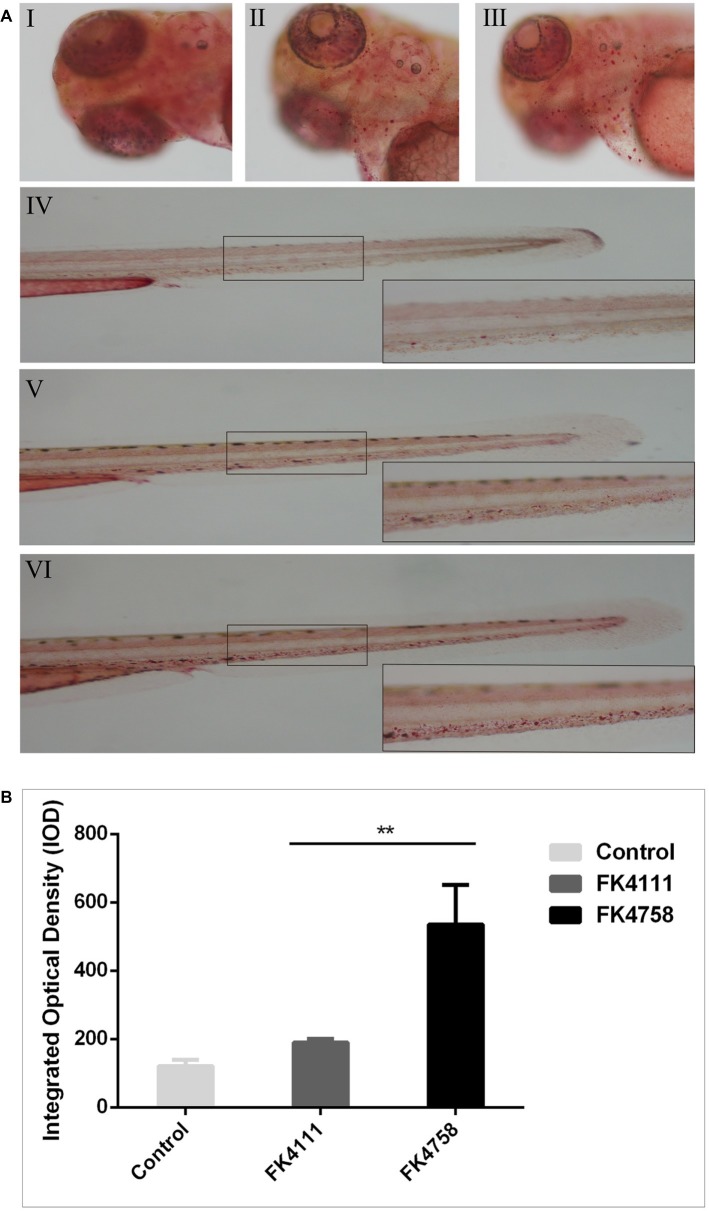
Evaluation of macrophages phagocytosis in zebrafish infected with FK4111 and FK4758. **(A)** Zebrafish larvae at 3 dpf were immersed in water containing bacteria with a final concentration of 6 × 10^8^ CFU/ml, collected at 8 hpi and analyzed by neutral red method (*n* = 10/group). Under the head of zebrafish, the bottom right corner (100×) was enlarged figure of the black box in the left (40×). (I, IV) Control group; (II, V) the group of FK4111 infection; (III, VI) the group of FK4758 infection. **(B)** Quantitative analysis of macrophages phagocytosis using integral optical density (IOD) by Image-Pro Plus image analysis software version 6.0. ^**^p < 0.01.

**Figure 5:**
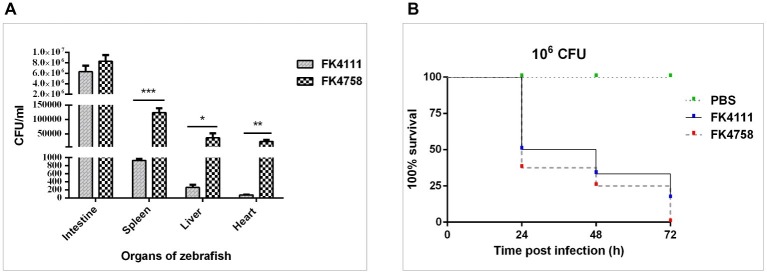
Assessment of bacterial burden of organs after intestinal colonization and analysis of the virulence of FK4111 and FK4758. **(A)** CFUs of bacteria cultured from the intestines, spleens, livers, and hearts of zebrafish exposed to FK4111 and FK4758 by immersion experiments. Data shown are the average CFUs cultured from 10 fish in each of the groups. ^*^, ^**^, and ^***^Represent *p* < 0.05, *p* < 0.01, and *p* < 0.001, respectively; **(B)** differences in the virulence of FK4111 and FK4758, established by the *Galleria mellonella* infection model.

As shown in Figure 4A (I, IV), there was no obvious red dot accumulation in the control group (Figure 4A; I, IV). The different degrees of aggregation of red spots after the immersion of the two colonizing bacteria in the intestinal tract indicated that the phagocytic activity of macrophages after infection was distinct (Figure 5A; II, V and III, VI). By counting the red dots of zebrafish in colonization group and control group, we found that the red dots formed by macrophage phagocytosis activity of strain FK4758 were more aggregated than those of the control group and strain FK4111 (*p* < 0.05). However, there was no significant difference in red dot aggregation (*p* > 0.1) between strain FK4111 and the control group, as obvious in Figure 4B.

### Organ Bacterial Load Assessment

We measured the bacterial load in the intestine, spleen, liver, and heart of adult zebrafish infected with bacterial suspension. The results showed that FK4111 and FK4758 had the highest bacterial load in the intestine as compared to that in other organs, count of 6.33 × 10^6^ and 8.33 10^6^ × CFU/ml, respectively, with no significant difference between them. However, in the spleen, liver, and heart, the FK4758 load was significantly higher than that of FK4111. The bacterial load of the two isolates in each organ is illustrated in Figure 5A.

### Analysis of the Virulence of Strains by *Galleria mellonella* Infection Model

As can be seen in Figure 5B, both isolates caused time-dependent death of larvae. No mortality was observed in the control which was injected with PBS. It is noteworthy that the survival rate of *G. mellonella* infected with FK4111 at the three time points was higher than that of FK4758 after the infection, indicating that the virulence of FK4758 was higher than that of FK4111. But there was no significant difference between the two groups (*p* > 0.05).

## Discussion

Since *K. pneumoniae* was first described, its multidrug-resistant and highly virulent strains have been reported many times. Importantly, more than 70% of the clinical carbapenem-resistant *Enterobacteriaceae* infections are caused by *K. pneumoniae* (Hennequin and Robin, 2016; Kanamori et al., 2017). Meanwhile, *K. pneumoniae* is an opportunistic pathogen that asymptomatically colonizes the human skin, mouth, respiratory tract, and gastrointestinal tract, becoming a potential host for infection. The emergence of these phenomena was undoubtedly considered a potential threat for global health. Some studies have shown that intestinal colonization by *K. pneumoniae* was strongly associated with its subsequent infection, which was related to the characteristics of colonization strain, including virulence, ST type, and carbapenemase phenotype (Wiener-Well et al., 2010; Hennequin et al., 2012). Despite its high clinical relevance, no clear distinction is available of the differences of the strains that cause infection after colonization and those with only asymptomatic colonization.

A total number of 11 MDR *K. pneumoniae* strains were isolated from the hospitalized patient. Among these strains, the non-carbapenem-producing strain FK4111 belonged to the ST37 type, while the carbapenem-producing strain FK4758 belonged to the ST11 type. These two strains were isolated from fecal samples. During this period, the ST37 strain was not detected in the blood since the detection of FK4111 colonization in the intestine, but the ST11 strain was identified in the blood after detection of FK4758 colonization in the intestine for 10 days. Therefore, there is reason to suspect that strain FK4758 (ST11) can cause blood infection, while strain FK4111 (ST37) might not cause a significant immune response. To investigate whether infections are different after colonization and evaluate the virulence of two colonization strains, zebrafish was employed as a host animal model to study the infection of *K. pneumoniae* and *G. mellonella* was used as a virulence evaluation model of strains. We found significant differences between the infection and organ metastasis caused by the two colonization strains in the zebrafish model. These results also revealed that the ST genotype and carbapenemase phenotype are closely related to virulence and post-colonization infection. To our knowledge, various risk factors for infection with *K. pneumoniae* have been identified, for instance underlying diseases and hospital stays etc. factors, but to our best acknowledge, the reports about risk factors specifically associated with progression from colonization to infection is rare.

In recent years, studies on clinical and subclinical infections have been successfully carried out by optimizing intramuscular injection in adult zebrafish; the effectiveness of antibiotics *in vivo* has also been investigated (Cheepurupalli et al., 2017). It is feasible to evaluate the virulence, neutrophil recruitment, bacterial clearance, and colonization of the bacteria by different methods of infection (injection/immersion) (Marcoleta et al., 2018). In this research, we used the specific immune mechanisms of zebrafish larvae (migration and aggregation of macrophages and neutrophils) to study the subsequent infection. We found no significant difference in the number of black granules between the FK4111 and the control group, but a significant difference existed in the black granules between FK4111 and FK4758. It is worth noting that the red dots formed by phagocytosis also followed the same pattern. In addition, as shown in Supplementary Figure S2, notable up-regulation of IL-1β was observed in spleen and liver tissues of zebrafish infected with FK4758 compared to the FK4111-infected group at 24 hpi. TNF-α was up-regulated in spleen and heart in zebrafish infected with FK4758 compared to the FK4111-infected group at 24 hpi. The neutrophil recruitment factor (IL-8) showed significant expression enhancement in multiple tissues of zebrafish in the late stage of FK4758 infection (12–24 h), and the expression level was significantly higher than that of the control group and FK4111. The results of Xiaohong Liu et al. showed that neutrophil chemokines were significantly up-regulated in the late stage of infection compared with the control group, which was similar with the results of this study (Liu et al., 2014). These results suggest that FK4758 (ST11) has a strong infection potential after its intestinal colonization of zebrafish larvae. However, FK4111 (ST37) did not induce an obvious immune process after the infection, and it was inferred that FK4111 only asymptomatic colonization in the intestine. We speculated that there was correlation between the ST types and the immune response induced by the infection caused after colonization. This provides a better direction for studies on the subsequent infection.

Meanwhile, to more accurately understand whether the two strains of bacteria induced systemic infection after their intestinal colonization, we measured the bacterial load of each organ of adult zebrafish. We found that both of them were successfully colonized in the intestine, with slight differences between the loads of two strains of bacteria in the intestinal tissue. Statistically significant differences were detected among the bacterial loads in spleen, liver, heart, and other organs. The FK4758 strain spread to many organs and eventually caused systemic infection. However, the FK4111 strain is rarely transferred to other organs, and this colonization does not cause a significant immune response, which was consistent with the difference in the immune response of the zebrafish larvae. These results suggested that FK4758 induced infection after colonization, but FK4111 did not.

In the present study, the mortality of the larvae was lower in the FK4111 isolates compared to FK4758 strain. Different *G. mellonella* survival rates were established in the two strains of *K. pneumoniae* isolates, probably due to the difference in the dynamics of the infection by the two strains (Pereira et al., 2015). However, the difference in the virulence was by no means unilateral. Successful infection may depend on many factors, including colonization, activation, or inhibition of the host immune system, and the production of potential toxins by the strains themselves.

Our research has the following strengths. First, we established a suitable animal infection model to compare the virulence and infection mechanism of *K. pneumoniae*. Second, the neutral red staining and SBB staining clearly reflected the immune response of zebrafish larvae, and the process was simple and rapid. Third, to simulate the colonization of zebrafish in a natural environment, we optimized the infection mode and concentrations previously reported, and adopted an appropriate concentration using the bacterial suspension immersion method to colonize zebrafish.

Nevertheless, some limitations of the present study also have to be acknowledged. First, neutral red and SBB staining cannot respond to the changes of immune response in real time. Second, after colonizing the zebrafish intestine, we could not control bacterial concentration that colonized the intestine. For this reason, we expanded the parallel sample size of colonized zebrafish larvae to ensure an appropriate accuracy of the experimental results.

In summary, our results showed that not all intestinal colonization of *K. pneumoniae* can lead to subsequent infection, the infection potential of *K. pneumoniae* after colonization is different. We also suggest that alternative host models of zebrafish and *G. mellonella* provide some advantages for studying the pathogenesis and virulence of *K. pneumoniae*. Prompt us should screen for colonization bacteria and also need to look for targeted drugs. At the same time, the admission of *K. pneumoniae* colonized patients should be examined strictly and effectively, so as to controlling the source of infection.

## Data Availability Statement

The raw data supporting the conclusions of this article will be made available by the authors, without undue reservation, to any qualified researcher.

## Ethics Statement

The animal study was reviewed and approved by Institutional Animal Care and Use Committee (IACUC) at Wenzhou Medical University.

## Author Contributions

XZ, TZ, and JC designed the study. XZ, YZhao, QW, JLin, RF, WB, GD, JLi, and YZhang performed the research and data analysis. XZ wrote the manuscript. TZ and JC supervised the research. All authors read and approved the final version of the manuscript.

### Conflict of Interest

The authors declare that the research was conducted in the absence of any commercial or financial relationships that could be construed as a potential conflict of interest.

## Footnotes

^1^http://bigsdb.pasteur.fr/klebsiella/klebsiella.html
