# Supplementary material for: Zebrafish and Galleria mellonella: Models to Identify the Subsequent Infection and Evaluate the Immunological Differences in Different Klebsiella pneumoniae Intestinal Colonization Strains
Source: Front Microbiol. 2019 Dec 2;10:2750. doi: 10.3389/fmicb.2019.02750 (PMC6900958; doi:10.3389/fmicb.2019.02750)
Supplement: Supplementary file 1 [file Data_Sheet_1.pdf]

Supplementary Material

1 Supplementary Figures

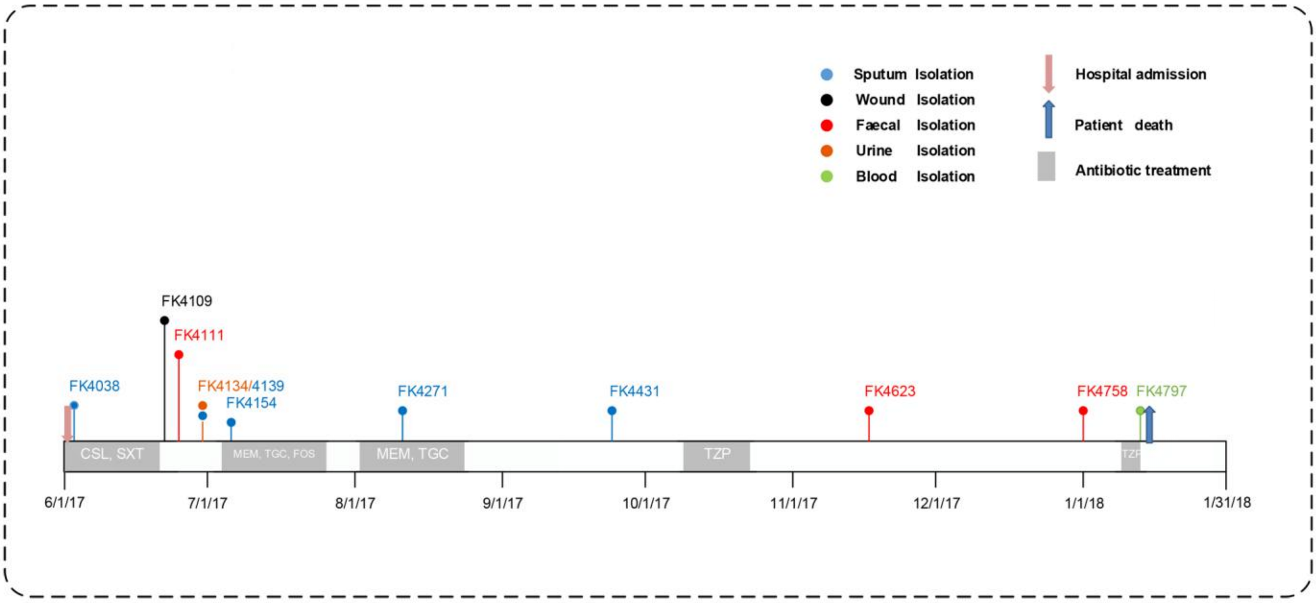

Supplementary Figure 1. Timeline of the isolated strains, antibiotics administered and clinical events during the hospital course. The major medical events that occurred during the seven-mouth period are shown. The bacteria isolated from different specimen types are labeled with different colored lines. The duration and types of the antibiotics used for treatment is also presented. CSL: Cefperazone-Sulbactam, SXT: Dispersibleco-trimoxazole, MEM: Meropenem, TGC: Tigecycline, FOS: Fosfomycin, TZP: Piperacillin and Tazobactam.

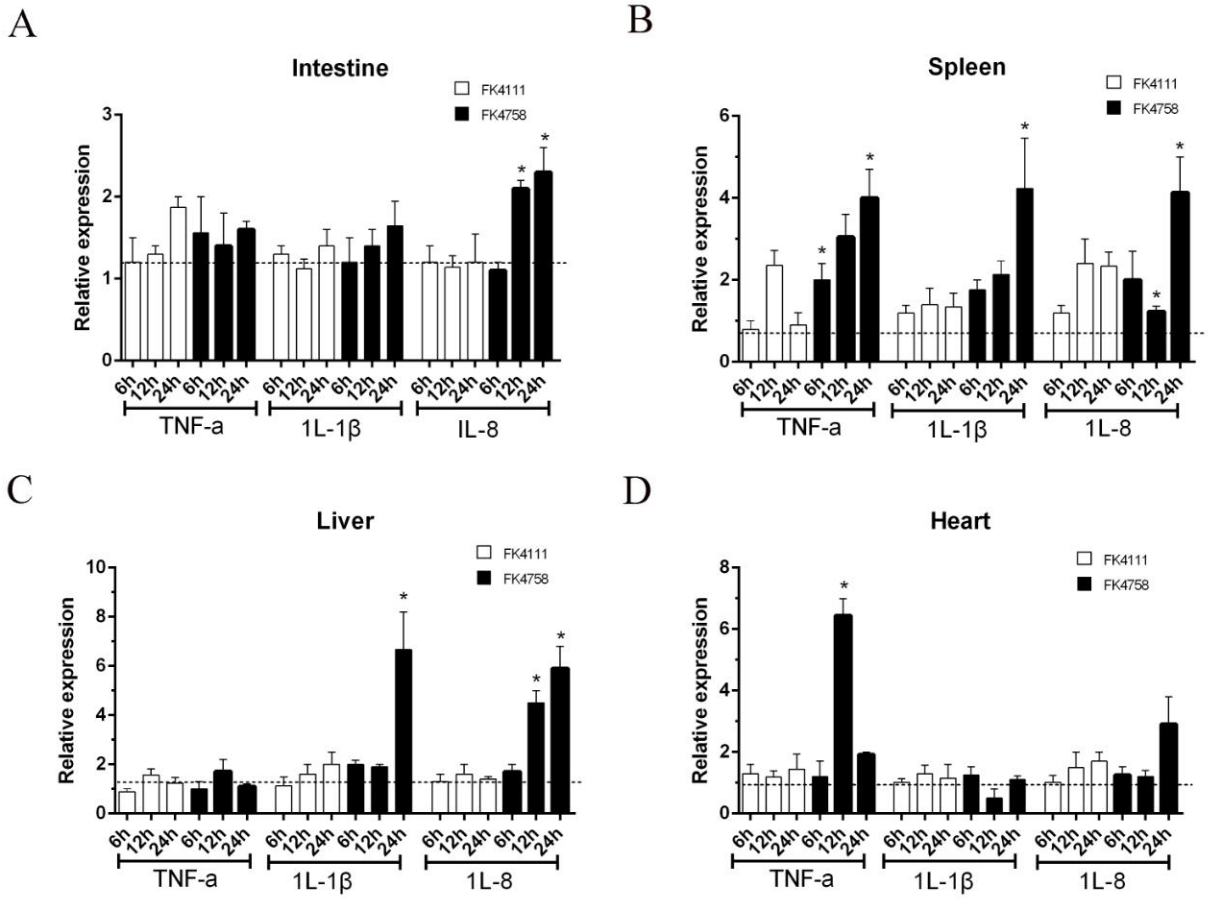

Supplementary Figure 2. Expressions of acute inflammatory response-related genes and antimicrobial response-related genes in zebrafish after *Klebsiella pneumoniae* infection. Healthy zebrafish were infected in bacterial suspension or mock-infected in PBS. Tissues from ten zebrafish of each group were sampled at 6, 12, 24 hpi. mRNA level of each gene was normalized to that of β-actin and relative expression was calculated by dividing the values of the infected tissues by those of the controls. Bars represented the mean relative expression of three individual replicates and error bars represented standard deviation. Statistical significance was analyzed between the FK4111-infected and FK4758-infected groups of zebrafish (*$P < 0.05$).
